# Beyond glucose: wearable and implantable biosensors for continuous monitoring of metabolic, hormonal, and inflammatory biomarkers in personalized cardiometabolic care

**DOI:** 10.3389/fbioe.2026.1885022

**Published:** 2026-07-08

**Authors:** Hong Cai, Yehui He, Chaoyue Wang, Yiqun Zhao, Chunhui Yang

**Affiliations:** 1 Department of Clinical Laboratory, The Second Hospital of Dalian Medical University, Dalian, Liaoning, China; 2 Information Center, The Second Hospital of Dalian Medical University, Dalian, Liaoning, China

**Keywords:** cardiometabolic care, implantable biosensors, inflammatory proteins, microneedles, multimodal sensing, wearable biosensors

## Abstract

Wearable and implantable biosensors are shifting biochemical assessment from episodic laboratory testing to continuous, context-rich physiological monitoring. Glucose remains the most successful translational model because continuous glucose monitoring has already established a credible pathway for analytical validation, clinical adoption, reimbursement, and regulatory approval. Yet cardiometabolic disease is not a single-analyte disorder. Insulin resistance, obesity, hypertension, heart failure, chronic kidney disease, stress-axis dysregulation, and chronic low-grade inflammation arise from interacting metabolic, endocrine, neural, and immune pathways. The next phase of the field therefore depends on whether biosensors can move beyond glucose while remaining biologically interpretable and clinically actionable. In this narrative review, we argue that personalized cardiometabolic care is the most attractive entry point for next-generation wearable and implantable biosensors because it combines high disease burden, dynamic physiology, rich biomarker biology, and an already established translational archetype. We integrate two complementary perspectives: a biomarker-centered framework that prioritizes glucose, lactate, ketones, uric acid, electrolytes, renal metabolites, cortisol, catecholamines, cytokines, acute-phase proteins, and selected cardiac markers; and a device-centered framework that examines sweat patches, microneedle-enabled interstitial fluid sensors, implantable systems, smart textiles, oral and ocular platforms, and hybrid closed-loop architectures. Particular emphasis is placed on biomarker-biofluid-device matching, the distinction between laboratory equivalence and continuous phenotyping, and the engineering barriers that determine real-world success, including partitioning across biofluids, skin-device coupling, biofouling, foreign-body response, calibration drift, reversibility of affinity-based sensing, power management, and multimodal data interpretation. We further discuss materials and interface strategies such as laser-induced graphene, conductive polymers, carbon nanomaterials, metal-organic frameworks, anti-fouling hydrogels, zwitterionic coatings, and drug-eluting surfaces; disease applications spanning diabetes, metabolic syndrome, heart failure, cardio-renal disease, and diabetic wound care; and the analytical, regulatory, and human-factor requirements for clinical adoption. The central message is that beyond-glucose biosensing will succeed only if it remains glucose-informed: glucose should serve as the translational backbone onto which additional metabolic, hormonal, inflammatory, and cardiovascular signals are layered according to biological kinetics, matrix suitability, and clinical decision need.

## 1 Introduction

Healthcare is undergoing a gradual but important transition from intermittent biochemical measurement toward continuous physiological observation (Ates et al., 2022; Kim et al., 2019). Traditional clinical testing relies on episodic sampling, most commonly venous blood followed by central laboratory analysis (Ray et al., 2019). That model remains indispensable for accuracy, standardization, and broad-panel testing (Ates et al., 2022; Kim et al., 2019; Ray et al., 2019), but it is intrinsically sparse (Brasier et al., 2024). Many clinically relevant molecular signals fluctuate over minutes, hours, and circadian cycles rather than remaining stable between clinic visits. As a result, conventional testing compresses dynamic physiology into a few isolated values and can miss excursions, recovery trajectories, and person-specific baselines. Continuous biosensing promises to fill this temporal gap by linking chemistry to lived physiology.

Wearable and implantable biosensors have become central to this shift because they can access body-associated biofluids repeatedly or continuously under real-world conditions (Kim et al., 2019). Their value lies not only in miniaturization, but also in the possibility of pairing biochemical signals with context such as physical activity, temperature, sleep, meals, medication timing, and symptoms (Ray et al., 2019). Unlike single-point diagnostics, these systems can capture trajectories, rates of change, coupling relationships, variability, and resilience (Brasier et al., 2024). Such information is particularly valuable in chronic cardiometabolic diseases, where deterioration is often progressive but decompensation may be abrupt. Diabetes, obesity, insulin resistance, hypertension, heart failure, chronic kidney disease, and cardio-renal syndromes all involve interacting disturbances in substrate handling, endocrine signaling, autonomic tone, fluid balance, tissue perfusion, and low-grade inflammation (Abreu et al., 2026).

The strongest current case for next-generation biosensors is therefore found in cardiometabolic medicine. Glucose monitoring has already demonstrated that continuous molecular sensing can reach routine care (Dehennis et al., 2015; Christiansen et al., 2018). Long-wear implantable and minimally invasive continuous glucose monitoring systems established not only technical feasibility, but also a translational template involving user acceptance, clinician familiarity, reimbursement logic, regulatory pathways, and digital integration (Renard et al., 2022). Long-wear implantable continuous glucose monitoring systems have progressed from 90-day to 365-day sensing paradigms, with multicenter clinical studies demonstrating acceptable accuracy and safety over extended implantation periods. In parallel, automated insulin delivery systems increasingly rely on CGM data streams, control algorithms, and insulin pumps as an integrated therapeutic architecture. These developments underscore that continuous sensing is now part of a connected therapeutic ecosystem rather than a stand-alone detector (Christiansen et al., 2018; Brown et al., 2019; Bailey et al., 2025).

**FIGURE 1:**
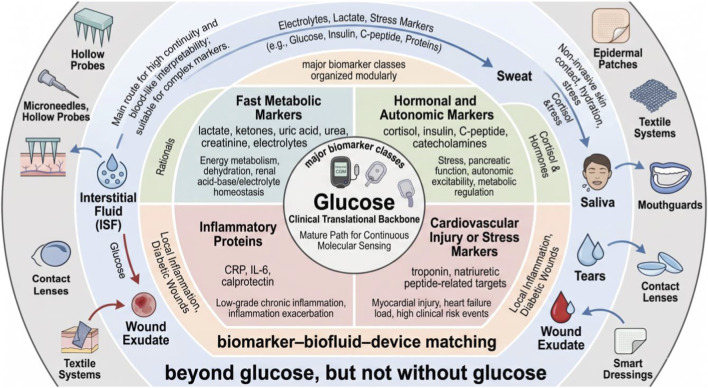
Biomarker–biofluid–device matching framework for beyond-glucose wearable and implantable biosensing in personalized cardiometabolic care. Schematic representation of a biomarker-centered framework for the rational design of next-generation wearable and implantable biosensors in cardiometabolic monitoring. Glucose is positioned as the translational backbone because continuous glucose monitoring has established a mature clinical, technological, regulatory, and digital-integration pathway for continuous molecular sensing. Beyond glucose, clinically relevant analytes are categorized into fast metabolic markers, including lactate, ketones, uric acid, urea, creatinine, and electrolytes; hormonal and autonomic markers, including cortisol, insulin, C-peptide, and catecholamines; inflammatory proteins, including C-reactive protein, interleukin-6, and calprotectin; and cardiovascular injury or stress markers, including troponin and natriuretic peptide-related targets. These biomarker classes are aligned with accessible biofluids, including interstitial fluid, sweat, saliva, tears, blood, and wound exudate, and with representative sensing architectures such as microneedles, hollow probes, epidermal patches, textile systems, mouthguards, contact lenses, implantable sensors, and smart dressings. The framework emphasizes that successful beyond-glucose biosensing depends on matching biomarker biology, biofluid accessibility, device architecture, and clinical decision context rather than simply expanding the number of detectable analytes. Abbreviations: CGM, continuous glucose monitoring; CRP, C-reactive protein; IL-6, interleukin-6; ISF, interstitial fluid.

Yet cardiometabolic disease is not a glucose-only problem. Glucose is a crucial anchor, but it does not fully describe metabolic flexibility, ketogenesis risk, stress-axis activity, inflammatory burden, or early tissue injury (Zhang et al., 2021; Wang B. et al., 2022; Chu et al., 2023). A broader biosensing framework is therefore needed. The central design problem is no longer only how to miniaturize sensors; it is how to match the right biomarker to the right biofluid, device architecture, transduction strategy, and clinical use case (Li J. et al., 2022; Li et al., 2024; Kim et al., 2024). Small, abundant, rapidly equilibrating analytes may be suitable for electrochemical sensing in sweat or interstitial fluid (Reza et al., 2024; Kim et al. (2017); Wang M. et al., 2022). Low-abundance hormones and proteins often require affinity recognition, signal amplification, regeneration strategies, or minimally invasive access to interstitial fluid (Wang B. et al., 2022; Friedel et al., 2023; Wang et al., 2021). This narrative review synthesizes the field through that translational lens and argues that cardiometabolic care provides one of the most coherent frameworks for beyond-glucose wearable and implantable biosensing (Figure 1).

## 2 Why cardiometabolic monitoring is a high-impact direction

Cardiometabolic monitoring is a particularly compelling application domain because it combines large disease burden, high clinical relevance, and molecular signals that change on timescales poorly captured by episodic laboratory testing (Global Burden of Cardiovascular Diseases and Risks, 2023 Collaborators, 2025). Diabetes, obesity, hypertension, heart failure, and chronic kidney disease create a sustained need for longitudinal monitoring rather than one-time diagnosis. In this context, biosensors are attractive not simply because they miniaturize measurement, but because they can reveal excursions, recovery kinetics, and patient-specific baselines that are invisible to sparse sampling (Tandon et al., 2024).

Second, cardiometabolic disorders are biomarker-rich (Abreu et al., 2026). The field already relies on a dense ecosystem of clinically meaningful analytes, including glucose, ketones, lactate, uric acid, electrolytes, insulin-related hormones, renal function markers, inflammatory mediators, natriuretic peptides, and cardiac injury proteins (Dervisevic et al., 2024). This enables biosensor science to be framed not merely as engineering miniaturization but as a form of dynamic laboratory medicine.

Third, cardiometabolic disease is fundamentally dynamic (Kjær et al., 2026; Weng et al., 2024). Postprandial excursions, exercise recovery, nocturnal hypoglycemia, dehydration, sympathetic surges, inflammatory flares, and early congestion often occur between clinical encounters (Shahub et al., 2025). These are precisely the kinds of events that intermittent testing captures poorly and continuous monitoring captures well.

Fourth, this area balances novelty with translational realism. A glucose-only framework is analytically mature but biologically incomplete (Sarswat et al., 2017), whereas platforms focused exclusively on niche neural analytes may be innovative yet clinically narrower (Fredj et al., 2025; Jayaraman et al., 2026; Fredj and Sawan, 2023). By contrast, beyond-glucose cardiometabolic biosensing preserves translational traction while still allowing real innovation through multiplexing, harder biomarkers, advanced materials, and new clinical algorithms (Dervisevic et al., 2025). Because the domain already has a validated anchor in glucose monitoring, expansion into adjacent biomarkers appears more credible than a *de novo* monitoring ecosystem (Dehennis et al., 2015; Li J. et al., 2022).

Finally, cardiometabolic monitoring aligns naturally with the broader trajectory of precision medicine (Ardalan et al., 2020; Eliasson et al., 2024). The goal is not only earlier diagnosis, but also continuous phenotyping, personalized intervention timing, risk forecasting, and treatment titration. A patient with diabetes, obesity, and hypertension may plausibly benefit from integrated monitoring of glucose excursions, ketone risk, hydration status, and cortisol rhythm in a way that no single intermittent test can provide (Coskun et al., 2025). Cardiometabolic biosensing therefore offers a persuasive vision of how wearable and implantable devices can evolve from impressive prototypes into clinically actionable infrastructure.

## 3 From intermittent testing to continuous phenotyping: a clinical laboratory perspective

The central laboratory remains the benchmark for analytical rigor. Standardized collection procedures, traceability, quality control, and established reference methods make laboratory testing indispensable for diagnosis and confirmation (Ates et al., 2022; Kim et al., 2019; Ray et al., 2019). However, wearable and implantable biosensors should not be judged only by whether they reproduce laboratory values point-for-point (Li J. et al., 2022; Li et al., 2024; Kim et al., 2024). Many biosensors sample different biological compartments, such as sweat, saliva, tears, or interstitial fluid rather than plasma (Arakawa et al., 2020; Park et al., 2018), and their value often lies in capturing trends, variability, rate of change, and context rather than absolute equivalence at a single timepoint. The future relationship between the laboratory and biosensor ecosystems is therefore complementary rather than competitive.

A useful translational framework is to distinguish four analytical scenarios. In the first, the biosensor measures an analyte in a compartment close to the clinical reference standard and seeks close agreement (Li J. et al., 2022). Continuous glucose monitoring in interstitial fluid approximates this model, although physiological lag still matters (Siegmund et al., 2017; Cappon et al., 2019). In the second, the sensor measures the same analyte in a different matrix, such as sweat glucose or salivary cortisol, where translational models are needed rather than direct equivalence (Chen et al., 2024; Liu et al., 2019). In the third, the device measures a correlated surrogate that still has clinical value, such as sweat sodium for hydration dynamics (Li J. et al., 2022; Choi et al., 2020). In the fourth, a device generates a composite phenotype by integrating multiple biochemical and physical signals even when no single analyte mirrors a conventional laboratory test (Ardalan et al., 2020). This final scenario may become especially important in cardiometabolic care, where the clinically useful output may be an event classifier, baseline deviation score, or predicted decompensation risk rather than a single concentration.

This perspective changes how biosensors should be validated. Limit of detection and short-term sensitivity are necessary, but they are not enough (Pérez and Orozco, 2022). What matters clinically includes drift over time, biological interpretability of the sampled matrix, resilience to motion and environmental changes, within-person reproducibility, and whether the resulting signal changes care (Cajigas et al., 2024; Ward, 2008). Continuous biosensing may ultimately extend laboratory medicine into the temporal domain by revealing excursions, coupling relationships, and resilience patterns that no central lab can observe on its own. At the same time, the field needs the conceptual discipline of laboratory medicine: careful matrix comparison, allowable error frameworks, interference studies, calibration strategies, and explicit links between signal behavior and clinical decisions (Pérez and Orozco, 2022).

The laboratory perspective also guards against a common conceptual mistake: treating all wearable outputs as if they should be interpreted like static diagnostic thresholds (Ardalan et al., 2020). In reality, continuous biosensors may deliver more value through within-person baselining and deviation analysis than through universal cutoffs. A chronic low-grade rise in an inflammatory signal, a blunted lactate recovery after exertion, or a widening gap between circadian timing and cortisol recovery may each be meaningful even when absolute values remain within traditional ranges (Asadian et al., 2025; Ganguly et al., 2021). This emphasis on trajectories is especially relevant in cardiometabolic medicine, where deterioration is often progressive and patterned rather than abrupt and binary.

## 4 Biomarker logic for personalized cardiometabolic care

A biomarker-centered review should begin with biology rather than hardware. The most persuasive beyond-glucose framework is not a list of everything that can be sensed, but a hierarchy of analytes that are dynamically informative, clinically actionable, and technically plausible. For cardiometabolic care, biomarkers can be grouped into four broad classes: fast metabolic markers; renal-metabolic and electrolyte markers; hormones and neurotransmitters; and inflammatory or tissue-injury proteins (Liu et al., 2024). Each class differs in abundance, kinetics, compartmentalization, reversibility requirements, and interpretive burden (Pérez and Orozco, 2022).

### 4.1 Glucose as the translational foundation

Glucose remains the foundation of the field because it satisfies a rare combination of properties. It has clear actionability, wide concentration dynamics, mature enzyme chemistry, and a large clinical ecosystem built around metrics such as time in range, hypoglycemia burden, and glycemic variability (Lv et al., 2025). Continuous glucose monitoring has already shown that molecular sensing can alter behavior, improve decision-making, and support closed-loop therapeutics. For this reason, glucose should remain the translational backbone of next-generation biosensing rather than being treated as a mature topic to leave behind.

At the same time, glucose is an incomplete descriptor of cardiometabolic physiology (Li V. L. et al., 2022). Glucose patterns alone do not distinguish insulin resistance from beta-cell failure, identify transitions toward ketogenesis, quantify exercise-related metabolic stress, or reflect stress-axis activation and inflammatory tone (Seidelmann et al., 2018). The correct move is therefore not to replace glucose, but to build beyond it. In practical terms, beyond-glucose biosensing should remain glucose-informed, with additional analytes layered onto glucose according to clinical context, biological rationale, and signal robustness (Dervisevic et al., 2025).

### 4.2 Fast metabolic markers: lactate, ketones, uric acid, urea, creatinine, and electrolytes

Lactate is among the most attractive next-step analytes because it bridges exercise physiology, tissue hypoperfusion, mitochondrial stress, sepsis, and heart failure (Sabatini et al., 2024). Continuous lactate monitoring may reveal exertional load, recovery kinetics, and impaired oxidative metabolism (Weng et al., 2024). Sweat-based lactate sensing is technologically mature, while microneedle-enabled interstitial fluid lactate monitoring may offer stronger clinical interpretability (Yang H. et al., 2024). The main limitation is that lactate is highly context dependent: local tissue metabolism, sweat rate, temperature, pH, and exercise state all affect readings. This makes lactate a particularly strong candidate for multimodal rather than standalone sensing (Hossain et al., 2024).

Ketone monitoring, especially beta-hydroxybutyrate, is highly relevant in insulin-deficient diabetes, fasting, ketogenic diets, critical illness, and metabolic flexibility assessment (Moya-Garzon et al., 2025). Glucose alone may not reveal a dangerous shift toward ketosis, particularly in settings such as sodium-glucose cotransporter-2 inhibitor use (Neuen et al., 2022). Continuous ketone sensing could therefore complement glucose in both acute safety monitoring and broader metabolic phenotyping. Recent studies suggest growing feasibility, but stable long-wear sensing chemistries and clinically interpretable thresholds still require development.

Uric acid, urea, and creatinine expand the field into renal-metabolic crosstalk (Sun et al., 2025; Hu et al., 2024). Uric acid is increasingly targeted in wearable platforms because of its relevance to purine metabolism and cardio-renal-metabolic risk (Yang et al., 2020; Li et al., 2023; Ding et al., 2026). Urea and creatinine remain classical renal markers, and their appearance in sweat and interstitial fluid makes them attractive for noninvasive or minimally invasive surveillance (Dervisevic et al., 2024; Yang H. et al., 2024; Hu et al., 2024). Although these analytes are not yet as mature as glucose or lactate for continuous monitoring, they are conceptually important because they position biosensors within a broader cardio-renal-metabolic framework rather than a diabetes-only framework.

Electrolytes, including sodium and potassium, are central to hydration, cardiovascular stability, arrhythmia risk, and exercise safety. Sweat sodium sensing is among the most mature noninvasive approaches and has obvious applications in sports physiology, occupational monitoring, and fluid balance (Gao et al., 2016). Potassium is more challenging because small analytical errors may have major clinical consequences (Welling et al., 2024). Electrolyte data are most useful when combined with contextual signals such as sweat rate, temperature, activity, and perhaps blood-pressure-related surrogates (Ardalan et al., 2020; Choi et al., 2020).

### 4.3 Hormones and neurotransmitters: difficult but high-value biomarkers

Hormones and neurotransmitters are analytically difficult but conceptually transformative. Cortisol is especially attractive because stress, sleep disruption, obesity, insulin resistance, depression, and hypertension all intersect with hypothalamic-pituitary-adrenal axis dysregulation (Fande et al., 2026). Continuous cortisol monitoring could help distinguish metabolic deterioration driven by lifestyle, circadian disruption, or psychosocial stress from deterioration driven by nutrition alone. Several wearable cortisol systems based on aptamers, field-effect transistors, impedance, or molecularly imprinted platforms now suggest that this goal is technically plausible, though still early (Torrente-Rodríguez et al., 2020; Wang B. et al., 2022; An et al., 2022; Singh et al., 2023; Garg et al., 2024).

Insulin and C-peptide are also high-value targets because they speak directly to beta-cell reserve, insulin resistance, and meal-related physiology (Chen et al., 2025). Their continuous or semi-continuous monitoring could eventually refine therapy selection and distinguish phenotypes that glucose patterns alone cannot separate. However, these analytes occur at far lower concentrations than glucose, often require affinity recognition, and demand careful interpretation of matrix lag and binding kinetics. Because insulin and C-peptide oscillate on meal-related timescales, the field will likely need not only better sensors, but also better models of postprandial dynamics and person-specific insulin sensitivity.

Catecholamines and related neurotransmitters, such as epinephrine, norepinephrine, dopamine, and serotonin, offer perhaps the most ambitious extension of cardiometabolic biosensing (Fredj et al., 2025; Jayaraman et al., 2026; Fredj and Sawan, 2023). These molecules report sympathetic activation, acute stress, and autonomic dynamics that influence blood pressure, heart rate, glycemic variability, and exercise recovery (Mugo et al., 2023). Their low abundance, pulsatile release, and susceptibility to interference make them difficult targets, but successful monitoring would bring wearable biosensing much closer to a genuinely integrative view of metabolism and stress biology.

### 4.4 Inflammatory and tissue-injury proteins

Inflammation is a defining layer of cardiometabolic disease. Low-grade inflammatory activity links obesity, insulin resistance, vascular dysfunction, and chronic tissue stress. Acute inflammatory surges also accompany infection, wound breakdown, and perioperative deterioration (Yang X. et al., 2024). This makes proteins such as C-reactive protein, interleukin-6, calprotectin, and selected chemokines attractive targets.

These biomarkers also expose the toughest analytical frontier. Proteins are larger, slower to diffuse, less abundant in skin-accessible fluids, and more prone to nonspecific adsorption than small metabolites (Pérez and Orozco, 2022; Cajigas et al., 2024). Their continuous monitoring therefore usually requires affinity capture, signal amplification, regeneration, and anti-fouling design (Kim et al., 2017). Still, recent progress in interstitial fluid protein sensing, wearable cytokine fabrics, and minimally invasive troponin monitoring suggests that the field is gradually moving from proof of concept to clinically interpretable trajectories (Park et al., 2025; Mirzajani et al., 2025). Selected cardiac markers, especially troponins and natriuretic peptides, are strategically important even if they remain less mature. Continuous or refreshable monitoring of tissue injury and hemodynamic stress could transform cardiovascular risk surveillance, heart failure decompensation detection, and peri-event triage.

### 4.5 Quantitative benchmarking: performance windows, drift, and wear duration

Recent reports indicate that beyond-glucose biosensing is no longer defined solely by proof-of-concept detection (Table 1). Instead, the field is beginning to resolve analyte-specific performance windows that matter for translation. For example, sweat lactate platforms have achieved a limit of detection (LOD) of 0.135 mM across a 0.1–50 mM range, while intradermal microneedle lactate patches have operated over an approximately 0.5–5 mM window that is more directly aligned with interstitial physiology (Reza et al., 2024; Weng et al., 2024). For cortisol, wearable platforms now span picomolar to nanomolar detection windows: an aptamer-TFT device reported a 1 pM LOD over 1 pM to 1 μM, and a wearable aptamer-FET array covered the physiological sweat/saliva range of 100 pM to 100 nM with second-scale response (Wang B. et al. (2022); An et al., 2022). For low-abundance proteins, a wearable IL-6 fabric sensor achieved a 280 fg/mL LOD with a working range of 1 pg/mL to 100 ng/mL, illustrating that analytical sensitivity is no longer the sole bottleneck for cytokine sensing (Chu et al., 2023).

**TABLE 1:** Clinically actionable biomarkers for wearable and implantable monitoring in personalized cardiometabolic care.

Biomarker (class)	Approximate physiological range in target matrix	Representative recent performance	Preferred matrix/Platform	Clinical relevance and key challenge	References
Glucose (fast metabolic)	ISF/plasma usually 3.9–10 mM in routine ambulatory states; larger excursions occur in diabetes	Commercial implantable CGM now supports up to 365-day wear; microneedle prototypes cover clinically relevant ISF windows (for example, 1–40 mM)	ISF; implantable CGM; microneedles	Highest actionability for titration and closed-loop care; challenge has shifted from raw sensitivity toward calibration stability, usability, and long-term tissue compatibility	Brown et al. (2019); Bailey et al. (2025); Yin et al. (2024); Tawakey et al. (2024)
Lactate (fast metabolic)	Plasma/ISF 0.5–2 mM at rest and may exceed 5 mM with intense exercise or hypoperfusion; sweat commonly rises into the low- to high-mM range	Sweat biosensor: LOD 0.135 mM and linear range 0.1–50 mM; intradermal microneedle patch: working range approximately 0.5–5 mM	Sweat and ISF; epidermal patches or microneedles	Useful for exercise load, recovery kinetics, metabolic stress, and perfusion context; challenge is strong dependence on activity, temperature, and local production	Reza et al. (2024); Weng et al. (2024)
Beta-hydroxybutyrate (fast metabolic)	ISF/blood typically 0.02–0.3 mM in the fed state, 0.5–3 mM during nutritional ketosis, and higher in marked ketosis	Commercial CKM can track 14-day BHB dynamics, but recent randomized data still showed progressive day-to-day decline consistent with drift	ISF; continuous ketone monitoring systems	Important for ketosis risk and metabolic flexibility; challenge is maintaining accuracy in the low baseline range where most ambulatory values cluster	Kjær et al. (2026)
Uric acid (renal-metabolic)	Serum usually 0.15–0.45 mM; sweat values are much lower, often in the low-μM to tens-of-μm range	Sweat dual-function sensor covered 0–40 μM uric acid; newer ISF microneedle platforms emphasize antifouling and lifetime prolongation	Sweat or ISF; wearable electrochemistry or microneedles	Links purine metabolism with gout and cardio-renal-metabolic burden; challenge is matrix comparability and long-term surface stability	Sun et al. (2025) Li et al. (2023)
Urea/creatinine (renal-metabolic)	Urea is typically 2.5–7.5 mM in blood/ISF; creatinine is usually 45–110 μM in blood, with lower, more variable concentrations in sweat	ISF urea microneedle patch reported a 3–18 mM linear range; wearable sweat platforms have also demonstrated simultaneous creatinine/uric-acid readout	ISF or sweat; microneedles, optical, or electrochemical wearables	Supports renal-metabolic surveillance and hydration assessment; challenge is sparse harmonized long-wear validation in real users	Dervisevic et al. (2024); Yang H. et al. (2024); Hu et al. (2024)
Sodium/potassium (electrolytes)	ISF/plasma Na+ 135–145 mM and K+ 3.5–5.0 mM; sweat Na+ commonly 10–70 mM and K+ 3–15 mM	Microneedle potentiometric platform calibrated over 5–200 mM Na+ and 1–100 mM K+; sensitivity RSDs were 3.12% (Na+) and 4.55% (K+) after repeated insertions	Sweat or ISF; potentiometric systems	Relevant to hydration, arrhythmia risk, and heart failure; challenge is rigorous calibration, especially for clinically consequential K+ measurements	Li et al. (2021)
Cortisol(endocrine)	Sweat and saliva usually contain low-pM to tens-of-nM free cortisol with marked circadian and stress dependence	Aptamer-TFT: 1 pM LOD, 1 pM-1 μM range; aptamer-FET: physiological 100 pM-100 nM window with second-scale response; 2026 flexible sensor: 0.58 ng/mL LOD and 0.5–150 ng/mL range	Sweat, saliva, or ISF; affinity-based wearables	Can report stress-axis dynamics and circadian disruption; challenge is low abundance, context dependence, and uncertain matrix-to-blood transfer	Wang B. et al. (2022); Fande et al. (2026); An et al. (2022)
Insulin/C-peptide (endocrine)	ISF insulin is typically in the tens-of-pm to low-nM range; C-peptide is generally sub-nM to low-nM and varies strongly after meals	Insulin microneedle biosensor quantified 0.01–4 nM with 94% accuracy; wearable C-peptide immunoassay supported at least 10 repeated assays per device	ISF; affinity-based minimally invasive systems	Potentially valuable for beta-cell reserve and insulin-resistance phenotyping; challenge is reversible recognition and durable protein-compatible sampling	Dervisevic et al. (2025); Chen et al. (2025)
Catecholamines (neurotransmitter)	Sweat/ISF concentrations are generally in the pM-nM range and can change rapidly during acute stress	On-body catecholamine monitoring remains largely proof-of-concept; robust long-wear quantitative benchmarks are not yet standardized	Sweat or ISF; advanced affinity or catalytic systems	Could reveal sympathetic activation and autonomic instability; challenge is very low abundance, pulsatility, and interference from structurally related molecules	Fredj et al. (2025); Jayaraman et al. (2026); Fredj and Sawan, (2023)
CRP/IL-6/calprotectin (inflammatory)	Sweat and ISF signals are often in the pg/mL-ng/mL range, whereas serum CRP is commonly reported in mg/L	Wearable IL-6 fabric sensor: 280 fg/mL LOD and 1 pg/ml-100 ng/mL range; perspiration-based CRP/IL-6/calprotectin panels have entered human feasibility studies	ISF, sweat, or wound fluid; affinity-based platforms	May help monitor chronic inflammation, wound deterioration, and acute decompensation; challenge is nonspecific adsorption, regeneration, and clinical thresholds	Chu et al. (2023); Shahub et al. (2025)
Troponin/natriuretic peptide-related targets (cardiovascular)	Circulating concentrations are typically in the pg/mL to low-ng/mL range; ISF transfer kinetics remain incompletely defined	Minimally invasive ISF troponin-I sensing has been demonstrated, but durable continuous outpatient benchmarking remains exploratory	ISF or minimally invasive sampling	Very high clinical value for injury and decompensation; challenge is ultra-low abundance combined with uncertain compartment kinetics	Mirzajani et al. (2025)

Equally important, emerging platforms clarify that durability is now a defining translational filter. Commercial implantable glucose sensing has reached 365-day wear under an FDA-cleared paradigm (Brown et al., 2019; Bailey et al., 2025), whereas a randomized continuous ketone monitoring study still observed a progressive day-to-day decline in interstitial beta-hydroxybutyrate output over 14 days, consistent with unresolved drift (Kjær et al., 2026). In interstitial fluid, an insulin microneedle biosensor quantified 0.01–4 nM with approximately 94% accuracy (Dervisevic et al., 2025), and a wearable C-peptide immunoassay introduced a regenerable format that supported at least 10 repeated assays per device (Chen et al., 2025). These comparisons underscore a central translational lesson: high sensitivity, broad dynamic range, low drift, and clinically useful wear duration do not mature simultaneously, and each biomarker therefore requires its own benchmark profile rather than being judged against glucose alone.

## 5 Biomarker-biofluid-device matching

The most important conceptual advance in beyond-glucose biosensing is the shift from device-centered classification to biomarker-biofluid-device matching. No biofluid is universally optimal, and no device format is universally appropriate (Li J. et al., 2022; Li et al., 2024; Kim et al., 2024). What matters is the fit between biomarker abundance, transport path, matrix composition, required continuity, user burden, and clinical decision purpose.

### 5.1 Interstitial fluid as the most credible minimally invasive matrix

Interstitial fluid currently offers the strongest compromise between biological relevance and patient acceptability (Abbasiasl et al., 2024). It is closer to blood than sweat or saliva, yet far easier to access repeatedly than venous blood. The success of continuous glucose monitoring established the practicality of subcutaneous and microneedle-enabled interstitial fluid sensing, and the same logic now underpins expansion toward lactate, ketones, urea, uric acid, electrolytes, insulin, C-peptide, drugs, and some protein targets (Brown et al., 2019; Bailey et al., 2025; Li J. et al., 2022; Dervisevic et al., 2025).

Interstitial fluid is not equivalent to plasma. Diffusion delays, local perfusion, tissue heterogeneity, inflammation, and device-induced perturbation all shape signal behavior (Hung et al., 2025). Nevertheless, for many hard biomarkers, especially those requiring blood-like interpretability, interstitial fluid is currently the most credible minimally invasive route. This is why many of the most sophisticated beyond-glucose platforms now rely on microneedles, hollow microprobes, on-needle sensing, or compact subcutaneous implants. The translational appeal of interstitial fluid lies not in perfect equivalence, but in acceptable approximation combined with high temporal density.

### 5.2 Sweat as the most user-friendly noninvasive matrix

Sweat remains highly attractive because it enables painless, skin-conformal, low-burden monitoring through epidermal patches, textiles, and passive or stimulated collection systems (Gao et al., 2016). It is especially useful for electrolytes, lactate, some metabolites, and selected stress-related targets. However, sweat must be interpreted mechanistically rather than treated as a simple surrogate for blood (Sato et al., 1989; Heikenfeld, 2016; Gao et al., 2023). It is not simply diluted blood. Gland physiology, flow-rate dependence, ductal reabsorption, evaporation, contamination, stimulation mode, and local skin conditions all influence concentration readouts (Pérez and Orozco, 2022; Sato et al., 1989).

For this reason, the most convincing sweat biosensors are no longer single-channel detectors (Halža et al., 2013). They are multiplexed or context-aware platforms that measure chemistry together with sweat rate, temperature, pH, motion, or time-resolved microfluidic history (Choi et al., 2020). This is especially important when the target analyte is a hormone or protein whose transport from blood to sweat is uncertain (Ji et al., 2026). In sweat-based beyond-glucose biosensing, the problem is often less whether a molecule can be detected than whether its signal can be interpreted honestly (Munje et al., 2017).

### 5.3 Saliva, tears, blood, and wound fluid as complementary niches

Saliva offers a noninvasive route for cortisol, uric acid, glucose, lactate, and drug-related analytes, especially in mouthguard formats (Sharma A. et al., 2024). Its advantages include repeated access and, for some targets such as cortisol, biologically meaningful reflection of free hormone dynamics (Fande et al., 2026). Its main limitations are oral biofouling, food contamination, pH shifts, and user burden (Kim et al., 2015; Kim et al., 2014). Tears remain attractive for ocular and glucose-linked applications through smart contact lenses (Elsherif et al., 2018; Tseng et al., 2018), but comfort, oxygen permeability, optical safety, and imperfect blood-tear relationships limit broader use.

Direct blood sensing remains analytically powerful but is difficult to reconcile with chronic outpatient wearability. Implantable or intravascular systems may be suitable in acute care or specialized monitoring, but thrombosis risk, infection, and user burden remain substantial barriers. Wound exudate, in contrast, is a highly relevant local fluid in diabetes and peripheral vascular disease (Gianino et al., 2018; Hu et al., 2025). Smart dressings that track pH, oxygen, glucose, uric acid, bacterial signatures, or inflammatory mediators illustrate how biosensing can be localized when local tissue chemistry is more informative than systemic sampling.

Not every biomarker needs the same route of access. The most realistic future ecosystem is layered (Li J. et al., 2022). Implantable or minimally invasive interstitial fluid systems should bear the greatest clinical load for analytes that require high continuity and blood-like interpretability (Facchi et al., 2025). Noninvasive sweat patches can supply complementary information on hydration, exercise, stress, or lower-resolution inflammation (Lin et al., 2022). Salivary or ocular devices may serve niche roles, while smart wound dressings address local tissue status (Arakawa et al., 2020). The coherence of the field improves when architectures are chosen according to biomarker biology and clinical task rather than according to an ideology of maximal noninvasiveness.

## 6 Device architectures and transduction strategies

The field now spans multiple form factors, each with distinct strengths and weaknesses. Epidermal patches are highly visible, comfortable, and compatible with sweat microfluidics, colorimetry, and electrochemical sensing (Yuan et al., 2025). Textile and skin-like electronics offer unobtrusive long-term use but face reproducibility challenges as garments shift, fold, and age (Abreu et al., 2026). Microneedle platforms are especially promising because they can penetrate the stratum corneum painlessly, access interstitial fluid, and support either fluid extraction or *in situ* sensing (Hung et al., 2025). Subcutaneous implantables provide the highest continuity but are limited by foreign-body response and replacement burden (Brown et al., 2019; Bailey et al., 2025; Ward, 2008). Hybrid closed-loop systems may eventually integrate biochemical sensing with therapeutic actuation or digital behavioral coaching.

Electrochemical sensing remains dominant because it is compact, sensitive, and compatible with low-power electronics (Nguyen et al., 2023; Teymourian et al., 2020). Amperometry works well for enzyme-linked metabolites such as glucose and lactate (Strambini et al., 2015). Potentiometry is effective for electrolytes and pH (Li et al., 2021). Voltammetry and impedance add flexibility for broader analyte classes, including several affinity-based designs (Wang B. et al., 2022; Fredj et al., 2025; Fredj and Sawan, 2023). Optical and fluorescence-based approaches are also important, particularly for implantable glucose sensing, wound sensing, and contact-lens readouts (Koh et al., 2016). Their advantages include reduced susceptibility to some electrical artifacts and the possibility of contactless or smartphone-based reading, although photostability and calibration remain concerns (Park et al., 2018; Elsherif et al., 2018).

The major expansion beyond glucose depends on affinity-based recognition. Hormones and proteins generally require antibodies, aptamers, molecularly imprinted polymers, or other synthetic receptors rather than classical oxidase chemistry (Parolo et al., 2020). Aptamers are especially attractive because binding-induced conformational changes can be transduced in field-effect transistor or electrochemical formats, including wearable cortisol sensing platforms (Wang B. et al., 2022; Singh et al., 2023). However, continuous affinity sensing imposes additional requirements: the recognition interface must be reversible, regenerable, or show manageable drift over time. This is one reason why proof-of-concept detection of a low-abundance analyte is much easier than durable continuous tracking of the same analyte on body (Li et al., 2017; Aleman et al., 2021; Fercher et al., 2021; Leung et al., 2021).

Microneedle systems deserve special emphasis because they have become a leading architecture for bridging laboratory-like relevance and wearable practicality (Fande et al., 2026). Integrated microneedle platforms have enabled continuous real-time monitoring of multiple analytes in interstitial fluid during daily activities, while affinity-enabled microneedle devices have extended this concept to continuous therapeutic-drug monitoring (Wu et al., 2022). Porous and other sampling-oriented microneedles further broaden the design space for ISF extraction and downstream sensing (Pang et al., 2024).

Representative multimetabolite platforms illustrate the value of combining analytes on a single wearable or minimally invasive device. Gao et al. (2016) integrated sweat glucose, lactate, sodium, and potassium sensors with temperature measurement and wireless transmission, establishing an early system-level model for multiplexed perspiration analysis. Li et al. (2021) extended multiplexing to interstitial fluid through a microneedle potentiometric array for simultaneous sodium and potassium monitoring. Wang B. et al. (2022) used regenerable graphene electrodes, molecularly imprinted recognition layers, iontophoretic sweat induction, microfluidics, and wireless electronics to monitor trace metabolites and nutrients during exercise and at rest. More recent platforms have combined urea and lactate or multiple sweat biomarkers within integrated microfluidic formats (Yang H. et al., 2024; Zhang et al., 2025). These studies show that multianalyte sensing is most informative when the selected markers have complementary kinetics and when shared sampling, calibration, and power architectures are designed as a unified system.

Multimodal signal fusion is increasingly a prerequisite rather than an enhancement. Sweat sodium is more interpretable when paired with sweat-loss dynamics, skin temperature, thermal flux, and motion; likewise, stress-related biochemical readouts such as sweat cortisol can be contextualized by simultaneous physiological metrics such as HRV (Gao et al., 2016; Ghaffari et al., 2021; Lin et al., 2024; Spinelli et al., 2025; Hossain et al., 2024). Accordingly, next-generation cardiometabolic and stress-monitoring platforms are moving toward biochemical-biophysical systems, and the clinically useful output may be a state estimate or risk classification rather than a concentration value alone (Evert et al., 2025).

Machine learning and artificial intelligence can further improve these platforms at three distinct levels: sensor-level correction of temperature, pH, motion, and drift; cross-channel data fusion for recognizing physiological states; and predictive analytics that convert trajectories into individualized risk estimates. Computationally assisted cortisol monitoring and AI-supported interpretation of continuous glucose data illustrate how algorithms can improve calibration and contextual interpretation (Liu et al., 2025; Ji et al., 2025). For multimetabolite systems, the most credible near-term role is not to replace analytical validation, but to integrate correlated biomarker and biophysical streams while quantifying uncertainty and preserving explainability (Abdelaal et al., 2024; Muse and Topol, 2024; Peng et al., 2026).

## 7 Materials and interface engineering for long-term operation

**FIGURE 2:**
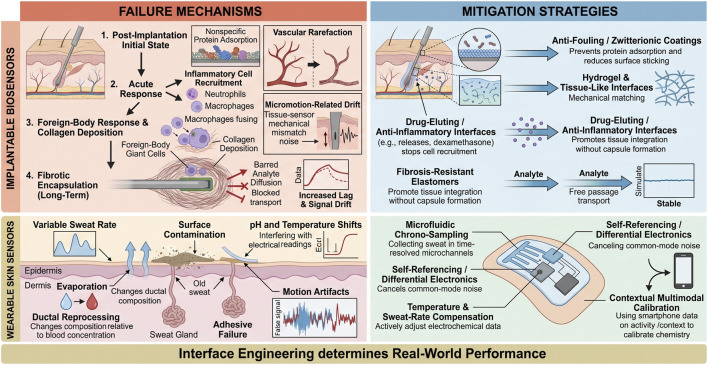
Interface-related failure mechanisms and mitigation strategies for wearable and implantable biosensors. Schematic overview of the major interface-driven barriers that limit the long-term accuracy, stability, and clinical reliability of wearable and implantable biosensors, together with representative engineering strategies for mitigation. In implantable biosensors, the post-implantation response begins with nonspecific protein adsorption and acute inflammatory cell recruitment, followed by macrophage activation, foreign-body giant cell formation, collagen deposition, vascular rarefaction, and long-term fibrotic encapsulation. These processes restrict analyte diffusion, alter local tissue physiology, increase signal lag, and promote calibration drift. Device–tissue micromotion can further introduce mechanical mismatch and signal noise. In wearable skin-mounted sensors, key sources of analytical error include variable sweat rate, evaporation, ductal reprocessing, surface contamination, pH- and temperature-dependent signal shifts, adhesive failure, and motion artifacts. These factors can distort electrochemical readouts and weaken physiological interpretability. Representative mitigation strategies include anti-fouling and zwitterionic coatings to reduce protein adsorption, hydrogel and tissue-like interfaces to improve mechanical matching, drug-eluting or anti-inflammatory designs to suppress cell recruitment, fibrosis-resistant elastomers to maintain analyte transport, microfluidic chrono-sampling to collect fresh sweat in a time-resolved manner, self-referencing or differential electronics to reduce common-mode noise, and contextual multimodal calibration using temperature, sweat-rate, activity, and smartphone-derived information. Collectively, these mechanisms highlight interface engineering as a central determinant of real-world biosensor performance.

Materials innovation has expanded the performance envelope of wearable and implantable biosensors, but the most important advance is not any single nanomaterial (Table 2; Figure 2) (Friedel et al., 2023). It is the emergence of fit-for-biomarker interface engineering. Mature analytes such as glucose benefit from optimized enzyme layers, mediator chemistry, and fluorescence-based implants (Yin et al., 2024). Electrolytes rely on selective membranes (Li et al., 2021). Lactate, urea, and uric acid can be approached using oxidases, nanozyme-enzyme hybrids, or catalytic surfaces (Reza et al., 2024; Dervisevic et al., 2024; Li et al., 2023). Hard biomarkers such as cortisol, insulin, or cytokines often require entirely different local microenvironments that stabilize affinity interactions, suppress nonspecific adsorption, and maintain signal reversibility (Wang B. et al., 2022).

**TABLE 2:** Key engineering and translational barriers with representative device or literature examples.

Barrier	Why it matters	Representative device or literature case	Quantitative lesson	Most relevant setting	References
Matrix mismatch	Sweat, saliva, and ISF do not map directly onto plasma and therefore cannot be validated by equivalence logic alone	Sweat cortisol platforms and the wearable IL-6 fabric sensor	pM to fg/mL sensitivity is impressive, but matrix-specific physiological ranges and transfer models remain essential for interpretation	All non-blood platforms	Wang B. et al. (2022); Chu et al. (2023); Fande et al. (2026)
Biofouling	Protein adsorption and surface contamination progressively degrade sensitivity and selectivity	Lifetime-prolonged uric-acid microneedles and antifouling interface strategies	Usable wear time is increasingly determined by interface engineering as much as by the transducer itself	Implantables; low-abundance analyte sensors	Sun et al. (2025); Jayakumar et al. (2023); Zhou et al. (2024)
Foreign-body response	Fibrotic encapsulation reduces analyte flux, changes local physiology, and drives long-term bias	Eversense implantable CGM systems and dexamethasone-acetate interface engineering	Clinically deployed implantables show that anti-inflammatory design can extend wear from months to 365 days	Subcutaneous implantables	Christiansen et al. (2018); Bailey et al. (2025); Brown et al. (2019)
Calibration drift	Long-term bias undermines trust even when short-term benchtop accuracy is strong	Continuous ketone monitoring study and Na+/K+ microneedle sensing	14-day BHB tracking still showed progressive decline, whereas Na+/K+ sensors retained sensitivity after repeated insertions with RSDs of 3.12% and 4.55%	All continuous systems	Kjær et al. (2026); Li et al. (2021)
Recognition reversibility	Affinity layers may saturate, degrade, or require regeneration during prolonged operation	Aptamer cortisol platforms and regenerable C-peptide immunoassay	Reversible aptamer systems can reach pM sensitivity, but protein monitoring may still require refreshable chemistry; one C-peptide device enabled at least 10 repeated assays	Hormones and proteins	Wang B. et al. (2022); An et al. (2022); Chen et al. (2025)
Environmental artifacts	Temperature, pH, sweat rate, motion, and local metabolism can distort the chemical signal	Sweat lactate systems and multimodal sweat/ISF wearables	mM-range linearity alone does not guarantee physiological specificity without contextual sensing	Wearables and epidermal patches	Kim et al., 2017; Weng et al. (2024); Asadian et al. (2025)
Power and telemetry	Sampling frequency, wireless transfer, and on-board processing are constrained by small form factors	Supercapacitor-powered lactate sensor and IoT perspiration patch	Energy strategy limits practical multiplexing and continuous use more than benchtop studies often imply	Wearables and hybrid systems	Ardalan et al. (2020); Asadian et al. (2025)
Clinical interpretability	A technically detectable signal may still have little decision value if thresholds and actions are undefined	Glucose as the 365-day benchmark versus ketone drift and cytokine ultrasensitivity	Analytical sensitivity, wear duration, and actionability mature on different timelines; biomarkers should be benchmarked separately rather than against glucose alone	All platforms	Bailey et al. (2025); Kjær et al. (2026); Chu et al. (2023)
Human factors	Comfort, visibility, insertion pain, replacement burden, and application burden strongly shape adherence	Implantable CGM reduces replacement frequency; textile and fabric systems emphasize breathability and comfort	A wearable that is analytically elegant but burdensome may fail in chronic disease care	Chronic disease use	Brown et al. (2019); Bailey et al. (2025); Chu et al. (2023)
Regulatory and reimbursement uncertainty	Undefined intended use slows adoption even when the underlying sensor is impressive	FDA-cleared iCGM pathway remains the strongest translation benchmark	Clear clinical claims and validated endpoints, not novelty alone, determine regulatory and reimbursement traction	Translation to practice	Brown et al. (2019); Bailey et al. (2025)
Matrix mismatch	Sweat, saliva, and ISF do not map directly onto plasma and therefore cannot be validated by equivalence logic alone	Sweat cortisol platforms and the wearable IL-6 fabric sensor	pM to fg/mL sensitivity is impressive, but matrix-specific physiological ranges and transfer models remain essential for interpretation	All non-blood platforms	Wang B. et al. (2022); Chu et al. (2023); Fande et al. (2026)

Flexible and stretchable substrates such as polyurethane, silicones, thermoplastic elastomers, and island-bridge layouts help reduce mechanical mismatch between the device and skin (Gao et al., 2023). Hydrogels are increasingly central because they preserve a hydrated interface, moderate mechanical stress, facilitate analyte diffusion, and can function as sensing matrices, anti-fouling barriers, or drug reservoirs (Abraham et al., 2018). Conductive polymers, graphene derivatives, carbon nanotubes, metal-organic frameworks, and hybrid nanostructures enhance surface area, charge transfer, and functionalization density (Reza et al., 2024; Fredj et al., 2025). In practical terms, these materials matter because they define whether a biosensor remains selective and stable in a messy biological environment rather than only in buffer.

Representative case studies clarify how material choices translate into measurable technological advances. In the fully integrated perspiration array of Gao et al. (2016), flexible electrochemical sensors, a temperature channel, wireless electronics, and skin-conformal packaging were co-designed to support multiplexed on-body analysis rather than isolated benchtop detection. Reza et al. (2024) functionalized polymer microneedles with reduced graphene oxide to increase electroactive surface area and enable continuous, wide-range lactate monitoring in interstitial fluid. For long-term implants, dexamethasone-acetate-containing interfaces in Eversense systems demonstrate how local anti-inflammatory material design can mitigate foreign-body effects and extend functional wear (Christiansen et al., 2018; Bailey et al., 2025). These examples indicate that the most useful material innovations directly address transport, mechanical matching, fouling, calibration stability, or tissue response.

For implantables, the interface is the dominant engineering problem (Jayakumar et al., 2023; Zhou et al., 2024). Biofouling begins immediately with protein adsorption and evolves into acute inflammation, macrophage recruitment, foreign-body giant cells, collagen deposition, and fibrotic capsule formation (Wang et al., 2015). These processes reduce analyte flux, impair reproducibility, and change local tissue physiology (Helton et al., 2011). Recent advances such as dexame-thasone-containing implantable sensors (Christiansen et al., 2018; Bailey et al., 2025), zwitterionic coatings (Jayakumar et al., 2023; Zhou et al., 2024), fibrosis-resistant elastomers (Zhou et al., 2024), self-cleaning thermoresponsive hydrogels (Abraham et al., 2018), and local drug-eluting strategies (Avula et al., 2013) suggest that the field is finally treating tissue engineering of the sensing interface as core design rather than an afterthought.

For wearables, the interface problem takes a different form. Skin-device coupling must remain robust despite sweat accumulation, drying, stretching, motion, and variable contact pressure (Ardalan et al., 2020; Choi et al., 2020; Gao et al., 2016). Surface contamination, pH shifts, and matrix evaporation also threaten accuracy (Pérez and Orozco, 2022; Sato et al., 1989). Here, material design intersects with system integration: microfluidic routing, adhesive mechanics, anti-fouling surface chemistry, and self-referencing channels all influence whether the measured chemistry reflects fresh sample, residual fluid, or pure artifact (Kim et al., 2017; Gao et al., 2016). Materials therefore matter not only because they improve sensitivity, but because they shape the truthfulness of the sampled biology (Ardalan et al., 2020; Choi et al., 2020; Pérez and Orozco, 2022).

## 8 Power, communication, and intelligent analytics

No biosensor becomes clinically useful through chemistry alone. System-level integration determines whether a promising transducer can survive beyond the benchtop (Lever et al., 2025; Chang et al., 2025). Wearable and implantable devices operate under severe constraints in power budget, data transmission, memory, and computational overhead (Zhang et al., 2022; Zhang et al., 2025). A sensor that performs excellently in short tests may fail in real use if battery life is poor, thermal load is excessive, or wireless transmission is unstable (Lever et al., 2025). This is particularly relevant for multiplexed cardiometabolic platforms, where several channels, environmental sensors, and on-board processing may need to coexist within a small form factor.

Low-power electronics, intermittent sampling strategies, and energy-aware design are therefore central to translation (Rong et al., 2021). Rechargeable batteries remain common in wearables, whereas implantables often depend on low-duty-cycle operation, inductive approaches, or highly efficient electronics (Yu et al., 2024). Energy harvesting through biofuel cells, triboelectric systems, supercapacitors, or thermoelectric mechanisms remains attractive, but practical deployment still depends on stability, miniaturization, and reliable energy density under realistic daily conditions. For most near-term cardiometabolic devices, the winning strategy may be intelligent power management rather than full self-powering.

Wireless interoperability is equally important. Modern biosensors are expected to communicate with smartphones, cloud systems, clinical dashboards, and, increasingly, therapeutic devices. Continuous glucose monitoring already showed that interoperability can transform a sensor from a monitoring device into the core of a therapeutic ecosystem (Khan et al., 2020). The same logic will apply to beyond-glucose systems. Ketone sensors may need to interact with insulin-delivery software, hydration-linked wearables may need to integrate with sports or occupational platforms, and heart-failure-oriented systems may need to feed remote monitoring dashboards. In each case, reliable connectivity and standardized data structures are as important as the transducer itself.

Artificial intelligence and advanced analytics are often invoked in this space, but their value should be framed carefully (Abdelaal et al., 2024). The strongest use cases are not abstract AI-enabled health, but specific tasks such as artifact removal, calibration transfer, drift compensation, multimodal pattern recognition, and within-person baselining (Muse and Topol, 2024). In cardiometabolic care, analytics may help distinguish exercise-induced lactate changes from pathological signals, separate meal-driven glucose excursions from stress-related changes, or integrate biochemical and physical data to predict loss of resilience before overt deterioration occurs (Park et al., 2026). The danger is that algorithmic sophistication may outpace clinical interpretability. A biosensor output that is highly accurate but opaque may be less useful than a slightly simpler signal that clinicians and patients can understand.

The likely future is therefore a layered analytics model. Raw signals will first be cleaned through device-level correction, then translated into biomarker trajectories, and finally interpreted in relation to behavior, environment, medication exposure, and personal history (Ji et al., 2025). In this model, intelligent analytics do not replace chemistry; they make chemistry clinically legible (Peng et al., 2026). The integration challenge is especially acute in cardiometabolic medicine, where the same biochemical pattern can reflect healthy adaptation, early decompensation, or simple context change depending on when and how it appears.

## 9 The hard problems: biofouling, drift, reversibility, and data interpretation

The gap between a proof-of-concept prototype and a clinically durable biosensor lies in the hard problems of long-term operation, especially biofouling, calibration drift, reversibility of recognition, and physiological interpretability of the signal (Li et al., 2017). In implantable systems, protein adsorption, foreign-body response, and the resulting mass-transport changes are well-established causes of signal attenuation and loss of long-term accuracy (Malone-Povolny et al., 2019). Noninvasive systems are not exempt: wearable sweat and saliva sensors also face matrix fouling, nonspecific adsorption, and performance degradation during repeated real-world use (Hossain et al., 2024).

Drift is not simply an engineering nuisance; it is a clinical problem because signal degradation can occur even when the device continues to output apparently stable numbers. Studies on electrochemical aptamer-based sensors have shown that long-term operation is limited by surface-layer degradation, matrix-dependent interference, and blood-component-induced drift, which is why meaningful validation must extend beyond short benchtop stability tests (Watkins et al., 2023).

Reversibility remains a defining bottleneck for hormones and proteins. Aptamer-based systems are attractive because binding-induced conformational changes can be transduced in a reversible manner, including in wearable cortisol sensors, but long-term performance can still deteriorate because of matrix effects and interface instability (Leung et al., 2021; Wang B. et al., 2022). Regenerable affinity and immunosensor formats broaden the range of accessible protein targets, yet regeneration itself is often difficult and may perturb the sensing interface, especially for high-avidity antibody-based systems (Quinn et al., 1999).

Finally, biochemical data are often ambiguous without context. Wearable studies of cortisol, lactate, sodium, and glucose increasingly show that interpretation depends on multimodal physiological information, circadian timing, sweat availability or sweat loss, exercise state, and subject-specific context rather than on concentration alone (Torrente-Rodríguez et al., 2020; Ghaffari et al., 2021; Lin et al., 2024; Spinelli et al., 2025). A useful synthesis from these studies is that continuous biosensing needs two calibrations: transducer calibration and physiological calibration. Long-term validation should therefore test not only whether the signal remains stable, but also whether the biological meaning of that signal remains stable in the same person over time.

## 10 Disease applications in personalized cardiometabolic care

**FIGURE 3:**
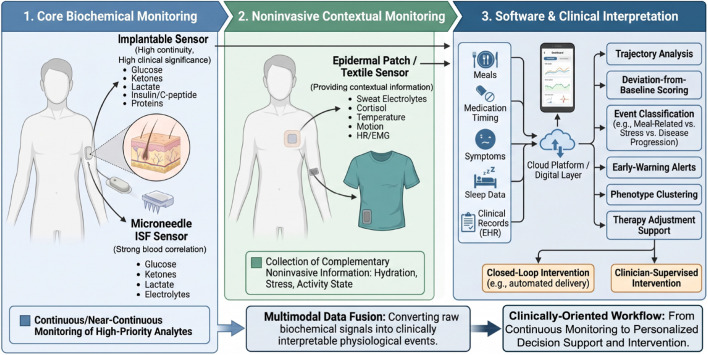
Multimodal workflow for continuous biosensing in personalized cardiometabolic management. Schematic workflow showing how continuous biochemical sensing, noninvasive contextual monitoring, and software-assisted interpretation can be integrated to support personalized cardiometabolic care. Implantable sensors and microneedle-based interstitial fluid devices provide continuous or near-continuous monitoring of high-priority cardiometabolic analytes, including glucose, ketones, lactate, electrolytes, insulin, C-peptide, and selected protein biomarkers. In parallel, epidermal patches and textile-based sensors collect complementary noninvasive information related to sweat electrolytes, cortisol, temperature, motion, heart rate, and electromyography-related activity. These biochemical and biophysical data streams are combined with patient-level contextual information, including meals, medication timing, symptoms, sleep patterns, physical activity, and electronic health records, through a cloud-based digital layer. Multimodal data fusion enables trajectory analysis, deviation-from-baseline scoring, event classification, early-warning alerts, phenotype clustering, and therapy adjustment support. This integrated workflow supports the transition of wearable and implantable biosensors from isolated analyte detectors to decision-support systems for risk stratification, early intervention, therapy titration, and closed-loop or clinician-supervised personalized management. Abbreviations: EHR, electronic health record; EMG, electromyography; HR, heart rate; ISF, interstitial fluid.

The translational value of beyond-glucose biosensing is best understood through clinical scenarios rather than individual analytes alone. In diabetes care, continuous glucose monitoring (CGM) remains the cornerstone, yet adjunctive biomarkers can significantly enhance risk stratification and personalization (Figure 3) (Cappon et al., 2019). Continuous ketone monitoring has emerged as a promising approach for identifying early transitions toward diabetic ketoacidosis and guiding timely intervention (Moonla S. et al., 2024). Lactate, as a dynamic metabolic intermediate, provides additional context for exercise response and systemic metabolic stress, particularly in physically active or critically ill individuals (Sharma et al., 2024). Looking ahead, real-time sensing of insulin and C-peptide may enable direct assessment of β-cell function versus insulin resistance, thereby refining disease phenotyping and informing individualized therapeutic strategies (Bonser and Garcia-Webb, 1984). These approaches may extend beyond type 1 diabetes to insulin-treated type 2 diabetes, gestational diabetes follow-up, and obesity-associated metabolic dysfunction.

In obesity, insulin resistance, and metabolic syndrome, reliance on single fasting measurements fails to capture the substantial heterogeneity of metabolic regulation. Large-scale studies have demonstrated that postprandial glycemic responses vary widely between individuals, even under standardized dietary conditions, underscoring the need for continuous and personalized monitoring (Zeevi et al., 2015). Continuous tracking of glucose excursions, stress-related hormones such as cortisol, and lactate dynamics can reveal underlying phenotypes of metabolic inflexibility, impaired recovery, or latent physiological burden (Bermingham et al., 2024). Importantly, the integration of multimodal physiological signals—such as heart rate variability, physical activity, and circadian timing—improves the interpretability of biochemical data and supports precision lifestyle interventions. In this context, continuous biosensing enables not only assessment of whether an intervention is effective, but also when it is effective and in which individuals.

Heart failure and cardio-renal syndromes represent another critical application domain. Early decompensation is often difficult to detect before the onset of overt clinical symptoms, contributing to high rates of hospitalization and readmission (Desai and Stevenson, 2012). Remote monitoring strategies, including implantable pressure sensors and wearable physiological devices, have demonstrated the potential to improve outcomes by enabling earlier intervention (Abraham et al., 2011). Future biosensing platforms that integrate electrolytes, hydration-related signals, renal metabolites, and natriuretic peptide surrogates may further enhance early detection and risk stratification beyond conventional weight and symptom monitoring. Within a remote-care framework, such systems could guide clinical decision-making by identifying patients who require urgent review, medication adjustment, or in-person evaluation.

Patients with chronic kidney disease (CKD) and hyperuricemia present a related opportunity for continuous biosensing. Current management relies heavily on episodic laboratory testing, which may fail to capture dynamic physiological instability (Levin et al., 2024). Continuous monitoring of urea, uric acid, electrolytes, and hydration status could provide earlier warning signals of disease progression or acute deterioration, particularly in patients with overlapping cardiometabolic conditions (Assalve et al., 2024). In this setting, biosensing is unlikely to replace standard laboratory diagnostics but may function as an early-warning layer that triggers confirmatory testing and timely clinical intervention.

Exercise physiology, rehabilitation, and preventive cardiology also stand to benefit substantially from continuous biosensing. Lactate is increasingly recognized not only as a marker of anaerobic metabolism but also as a signaling molecule that reflects systemic metabolic state and adaptation (Wang and Zhu, 2025). Continuous monitoring of lactate, glucose, sodium, and cortisol can improve assessment of training load, identify maladaptive stress responses, and guide individualized rehabilitation programs. Multimodal wearable systems that integrate biochemical sensing with physiological signals such as temperature, motion, and cardiovascular dynamics provide a more comprehensive picture of human performance and recovery (Gao et al., 2016). In these contexts, the key advantage of biosensing lies in its immediacy, translating otherwise invisible physiological processes into actionable feedback for both patients and clinicians. The same principles may support personalized lifestyle coaching and early intervention in high-risk but predisease populations.

Diabetic wound care illustrates a different but highly relevant use case (Yang Z. et al., 2024). Smart dressings capable of sensing pH, oxygen, glucose, uric acid, protease activity, bacterial metabolites, or inflammatory markers connect systemic cardiometabolic disease with local tissue failure (Wang et al., 2025). This reminds us that personalized monitoring is not restricted to systemic blood-like sampling (Yang Z. et al., 2024; Jiang et al., 2026). In some cases, local chemistry is the most clinically meaningful chemistry. Such wound-oriented platforms may become one of the earliest settings in which beyond-glucose biosensing affects outcomes through earlier detection of infection, ischemia, or stalled healing.

## 11 Clinical translation: what will determine real-world adoption?

Clinical adoption requires more than elegant materials, impressive sensitivity, or wireless readout (Brasier et al., 2024). The first requirement is choosing the right biomarker for the right question. Not every detectable molecule deserves continuous monitoring. A strong target should fluctuate in a clinically meaningful way, provide information poorly captured by intermittent testing, and plausibly influence decisions (Eliasson et al., 2024). Glucose clearly meets these criteria. Cortisol, ketones, selected inflammatory proteins, and hydration-linked electrolytes may do so in defined contexts (Wang B. et al., 2022). Some other analytes may be better suited to periodic rather than truly continuous wearable assessment.

The second requirement is analytical validation beyond limit of detection. Clinical translation demands drift studies, interference testing, calibration stability, environmental robustness, inter-user variability assessment, and a transparent description of how the sampled matrix relates to the clinical reference compartment (Pérez and Orozco, 2022; Cajigas et al., 2024). For sweat and saliva, this means demonstrating when concentration trends are informative even if direct plasma equivalence is weak (Fande et al., 2026). For interstitial fluid, it means defining lag, local heterogeneity, and calibration behavior under realistic physiology rather than idealized conditions (Li J. et al., 2022).

The third requirement is defining clinically meaningful endpoints. A biosensor signal is valuable only if it changes care or outcomes (Ardalan et al., 2020). Future studies must therefore move beyond simple correlation with laboratory assays and test whether biosensor-guided monitoring improves time in range, reduces severe excursions, predicts deterioration earlier, enhances adherence, or supports better titration decisions.

Human factors matter just as much. Chronic disease populations are already burdened by medications, appointments, and devices. Comfort, visibility, insertion pain, replacement frequency, app usability, skin tolerance, and confidence in the data all shape real-world uptake (Brown et al., 2019; Bailey et al., 2025; Ardalan et al., 2020). The translation literature in glucose monitoring already demonstrates that user trust and burden directly shape adherence; beyond-glucose platforms will face an even higher bar because their clinical value is not yet as culturally normalized (Lever et al., 2025; Chang et al., 2025).

Regulatory and reimbursement logic also require specificity. Glucose technologies succeeded because their intended use was clear and their performance metrics matched decision risk. Future cardiometabolic biosensors will need similarly well-defined claims. A device marketed for wellness occupies a very different regulatory space from one intended for medication titration or acute deterioration detection. Reimbursement will likely follow only when clinical and economic value are demonstrated. Finally, data governance and equity cannot be an afterthought, because systems that remain expensive, urban-centered, or smartphone-dependent risk widening disparities rather than reducing them.

## 12 Future directions

The next decade will likely be defined by several converging shifts. First, the field will move from single-analyte devices to selective multiplexing. The best platforms will not necessarily measure the most analytes, but the most complementary combinations. A glucose-plus-ketone platform, for example, may be more clinically useful than a technically impressive but weakly interpretable ten-marker patch.

Second, minimally invasive interstitial fluid access will become even more central. Microneedle-based systems occupy a promising middle ground between the interpretability of implantables and the user friendliness of noninvasive wearables. They may become the dominant architecture for monitoring difficult but clinically important biomarkers such as insulin, C-peptide, cytokines, or drug exposures.

Third, affinity-based sensing will expand the analyte repertoire, but only if paired with advances in reversibility and interface stability. This is where some of the most important innovation is likely to occur. The field does not merely need better binders; it needs recognition layers that remain trustworthy over time *in vivo*. Fourth, implantables will increasingly rely on tissue-responsive materials rather than passive tolerance of encapsulation. Anti-inflammatory, anti-fouling, and mechanics-aware designs are likely to become standard.

Fifth, biosensors will be integrated into algorithms and care pathways rather than functioning as isolated gadgets. Their outputs will feed remote monitoring systems, digital therapeutics, risk models, and eventually closed-loop or clinician-supervised intervention systems. Sixth, the conceptual goal of the field may broaden from disease control to health-state mapping. In cardiometabolic medicine, this means using longitudinal biochemical data to detect loss of resilience before conventional thresholds are crossed.

Such a shift would not replace the central laboratory; it would extend it. Central laboratories will continue to provide confirmation, standardization, and broad phenotyping, while wearable and implantable biosensors contribute temporal density, ecological validity, and action-triggering insight. The most successful future platforms will probably be those that integrate seamlessly with conventional care rather than those that claim total disruption.

## 13 Conclusion

Wearable and implantable biosensors are entering a decisive phase. The field has matured beyond asking whether on-body chemistry can be measured at all. The more important questions now concern which biomarkers deserve continuous monitoring, which biofluids provide interpretable access, which device architectures can operate reliably over meaningful timescales, and which signals can actually improve care. Personalized cardiometabolic monitoring provides a particularly strong framework in which to answer these questions because it combines rich biomarker biology, dynamic physiology, and a proven translational anchor in continuous glucose monitoring.

The most credible path forward is therefore beyond glucose, but not without glucose. Glucose should remain the validated backbone of the field, while additional metabolic, renal, endocrine, inflammatory, and cardiovascular markers are layered according to kinetics, matrix suitability, and decision need. Interstitial fluid will likely remain the principal route for clinically demanding continuous monitoring, while sweat, saliva, tears, and local wound fluids provide complementary context or specialized value. Success will depend not only on better chemistry and better materials, but also on analytical validation, anti-fouling and mechanics-aware interfaces, multimodal contextualization, human-centered design, and explicit clinical endpoints.

In that sense, the future of wearable and implantable biosensors is not a race to measure everything everywhere. It is a move toward analytically honest, biologically coherent, and clinically useful systems that turn continuous molecular information into better cardiometabolic care.
